# Building a Human Ovarian Antioxidant ceRNA Network “OvAnOx”: A Bioinformatic Perspective for Research on Redox-Related Ovarian Functions and Dysfunctions

**DOI:** 10.3390/antiox13091101

**Published:** 2024-09-12

**Authors:** Carla Tatone, Giovanna Di Emidio, Rosalia Battaglia, Cinzia Di Pietro

**Affiliations:** 1 Department of Life, Health and Experimental Sciences, University of L’Aquila, 67100 L’Aquila, Italy; carla.tatone@univaq.it (C.T.); giovanna.diemidio@univaq.it (G.D.E.); 2 Department of Biomedical and Biotechnological Sciences, Section of Biology and Genetics, University of Catania, 95123 Catania, Italy; dipietro@unict.it

**Keywords:** ceRNA network, oxidative stress, female fertility, antioxidant cell defense, non-coding RNAs

## Abstract

The ovary is a major determinant of female reproductive health. Ovarian functions are mainly related to the primordial follicle pool, which is gradually lost with aging. Ovarian aging and reproductive dysfunctions share oxidative stress as a common underlying mechanism. ROS signaling is essential for normal ovarian processes, yet it can contribute to various ovarian disorders when disrupted. Therefore, balance in the redox system is crucial for proper ovarian functions. In the present study, by focusing on mRNAs and ncRNAs described in the ovary and taking into account only validated ncRNA interactions, we built an ovarian antioxidant ceRNA network, named OvAnOx ceRNA, composed of 5 mRNAs (SOD1, SOD2, CAT, PRDX3, GR), 10 miRNAs and 5 lncRNAs (XIST, FGD5-AS1, MALAT1, NEAT1, SNHG1). Our bioinformatic analysis indicated that the components of OvAnOx ceRNA not only contribute to antioxidant defense but are also involved in other ovarian functions. Indeed, antioxidant enzymes encoded by mRNAs of OvAnOx ceRNA operate within a regulatory network that impacts ovarian reserve, follicular dynamics, and oocyte maturation in normal and pathological conditions. The OvAnOx ceRNA network represents a promising tool to unravel the complex dialog between redox potential and ovarian signaling pathways involved in reproductive health, aging, and diseases.

## 1. Introduction

The ovary is central to female reproductive function, providing oocytes for fertilization and synthesizing essential reproductive hormones. Primordial follicles formed during fetal development establish a finite reserve of primary oocytes, which lasts up to about 50 years in humans. During the fourth decade of life, the ovarian follicle pool declines, leading to a decrease in oocyte competence [1]. This process is known as ovarian aging and is responsible for the early decline in the reproductive function of women [2]. In addition to aging, ovarian function can be hampered by pathological conditions such as premature ovarian insufficiency (POI) and polycystic ovarian syndrome (PCOS) [3]. Both aging-related and pathological ovarian dysfunctions share oxidative stress as one of their main causative mechanisms [2,4,5,6]. This emphasizes the need for research to develop effective strategies based on selective targeting of specific redox-modulating mechanisms, especially considering the limited evidence in support of supplemental oral antioxidants for sub-fertile women [6,7].

Recent evidence has shown the involvement of non-coding RNAs (ncRNAs) in the antioxidant systems that scavenge free radicals to maintain a healthy level of reactive oxygen species (ROS). ROS are by-products of cellular oxidative metabolism and play a pivotal role in many cellular functions. Gene expression, cell signaling, and redox homeostasis all depend on the equilibrium between the creation and removal of ROS, known as “redox homeostasis” [8,9]. This is maintained by a highly responsive dynamic system that detects changes in redox status and realigns metabolic activities to restore stability [10]. Either an increase in ROS concentration or a decrease in scavenging capacity causes an imbalance in the redox environment, leading to ROS accumulation and oxidative damage to lipids, proteins, and DNA [8,9].

ncRNAs, which constitute most of the human transcriptome, perform essential regulatory functions at every step of gene expression [11]. They are classified into small non-coding RNAs (e.g., microRNAs), smaller than 200 nucleotides, and long non-coding RNAs (lncRNAs) ranging from 200 nucleotides to 100 kilobases or more [11]. LncRNAs, the most heterogeneous class, are involved in a wide spectrum of molecular mechanisms regulating genome functions, generating complex networks of RNA-RNA competitive interactions [12]. Different studies have demonstrated interactions among lncRNAs and miRNAs, miRNAs and mRNAs, and lncRNAs and mRNAs [12,13]. These RNA molecules collaborate to create dynamic regulatory networks, with lncRNAs acting as competing endogenous RNAs (ceRNAs) [14,15]. ceRNA networks are intricate, as multiple miRNAs can target a single mRNA, and one lncRNA can sponge various miRNAs, influencing different mRNAs.

ceRNAs are also strong proponents of many diseases [16,17,18]. Some ncRNAs can worsen disease progression by impacting ROS-related processes, while others can effectively protect cells from ROS-induced damage [19]. These RNAs also modulate gene expression within tissue-specific ceRNA networks [12,13], playing a crucial role in maintaining redox balance by affecting key antioxidant enzymes [20,21].

In this challenging new context, we aimed to investigate the potential regulation of antioxidant enzymes by ceRNA networks in the ovary. Using a bioinformatics approach, we developed a prediction model to explore interactions among mRNAs encoding antioxidant enzymes, miRNAs, and lncRNAs, focusing on RNAs known to be expressed in the human ovary. Based on this analysis, we built a potential ovarian antioxidant ceRNA network, here referred to as OvAnOx ceRNA. This network comprises miRNAs targeting antioxidant enzyme mRNAs and the lncRNAs targeting these miRNAs.

## 2. Materials and Methods

### 2.1. PICO Statement

This study was designed following the PICO framework and based on the following considerations:

Problem: are antioxidant enzymes regulated by ceRNA networks in the ovary?

Intervention: a bioinformatics approach is used to predict interactions among RNA molecules.

Comparison: interactions between miRNAs and antioxidant enzyme mRNAs and lncRNAs and miRNAs are compared using known databases and literature to ensure accuracy and relevance.

Outcome: prediction of an ovarian antioxidant ceRNA network, OvAnOx ceRNA, emerges as a promising tool to investigate the complex dialog between redox potential and ovarian signaling pathways involved in age-related or pathological ovarian dysfunction.

### 2.2. Gene Ontology and Pathway Analysis, Localization, and Expression of the 21 Antioxidant Enzymes

This study focused on 21 antioxidant genes [22]. The Gene Ontologies (GOs) enrichment analysis, including molecular functions, and Reactome pathways identification, for the 21 antioxidant enzymes, were performed using Panther 19.0 “http://www.pantherdb.org (accessed on 31 May 2024)”. The statistical overrepresentation test was executed. GOs and Reactome pathways with a *p*-value < 0.05 were chosen.

To verify their expression in the human ovary, we queried the Ovarian Kaleidoscope Database “https://appliedbioinfo.com/ (accessed on 3 June 2024)”. For cellular localization, we consulted scientific papers annotated in common databases. Mitochondrial localization was investigated using Human Mitocarta3.0 “https://www.broadinstitute.org/mitocarta/mitocarta30-inventory-mammalian-mitochondrial-proteins-and-pathways (accessed on 3 June 2024)” and MitoProteome “http://www.mitoproteome.org (accessed on 3 June 2024)”. Exosome localization was assessed using Exocarta “http://www.exocarta.org (accessed on 3 June 2024)” and exoRBase 2.0 “http://www.exorbase.org (accessed on 3 June 2024)” databases. Transcript expression in the human ovary was explored by querying the Human Protein Atlas (HPA) tissue dataset section https://www.proteinatlas.org (accessed on 10 June 2024).

### 2.3. Construction and Analysis of LncRNA-miRNA-mRNA Competing Endogenous RNA Networks

The miRNA-mRNA interaction analyses were performed using the miRTarbase “https://mirtarbase.cuhk.edu.cn (accessed on 20 June 2024)” database, selecting only interactions validated by functional experimental evidence. To investigate the interactions between lncRNAs and the miRNAs targeting antioxidant enzymes’ mRNAs, we consulted Starbase “https://starbase.sysu.edu.cn/ (accessed on 27 June 2024)”, selecting lncRNAs with at least two target binding sites for miRNAs.

We designed the competing endogenous RNA networks (ceRNA network) considering the miRNAs targeting antioxidant enzyme mRNAs and the lncRNAs targeting these miRNAs. The interaction networks of miRNA-mRNA, lncRNA-miRNA, and lncRNA-miRNA-mRNA were designed using Cytoscape 3.8.2, and the centrality parameters of individual nodes were calculated.

### 2.4. Cellular Localization of miRNAs and lncRNAs Regulating the Antioxidant Genes

In order to investigate if the previously identified miRNAs and lncRNAs are expressed in the ovary, we used miRDB “http://www.mirdb.org (accessed on 28 June 2024)” and lncBase v.3-DIANA tool “https://diana.e-ce.uth.gr/lncbasev3 (accessed on 28 June 2024)”, respectively.

## 3. Results

### 3.1. Antioxidant Genes Control Significant Molecular Functions and Biological Pathways

**Figure 1:**
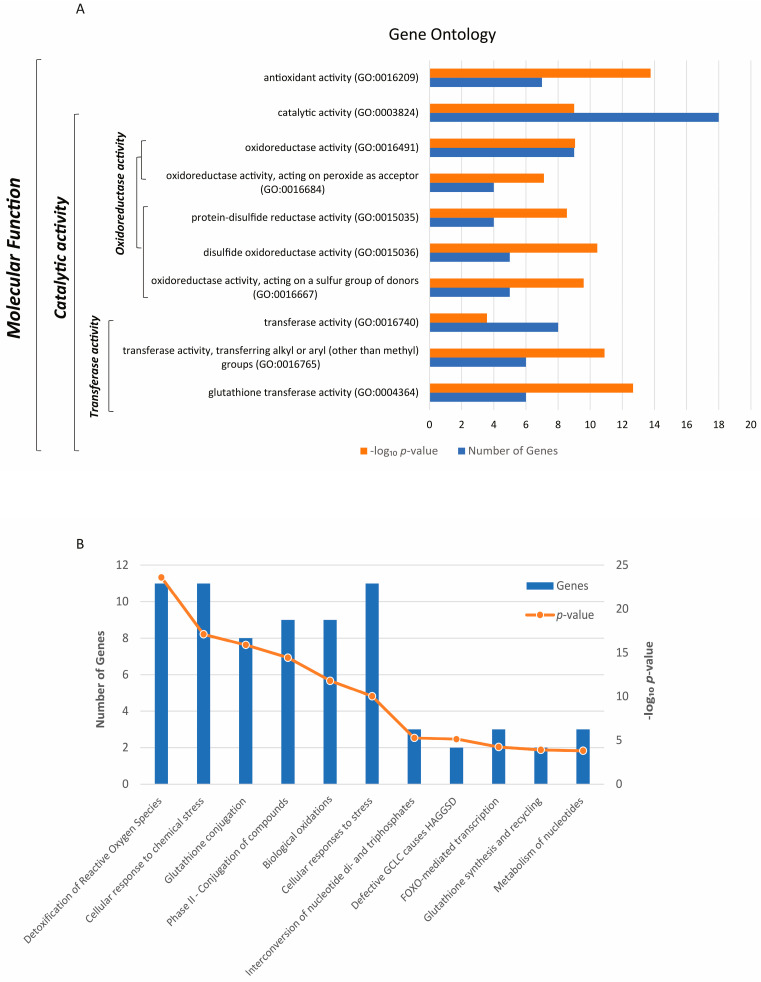
Molecular functions (GO) (**A**) and associated biological pathways (**B**) of the 21 antioxidant enzymes-encoding genes selected for this study. The significance is reported as a −log_10_ *p*-value for both panels (*p* < 0.05).

The Gene Ontology (GO) analysis revealed a significant enrichment of the 21 genes selected in this study across 10 molecular functions (Figure 1A). Molecular functions are listed hierarchically from top to bottom, with each gene transcript potentially associated with multiple functions. Catalytic activity (GO:0003824) emerged as the predominant GO category, with a high number of genes contributing (18), and encompassing functions such as oxidoreductase activity (GO:0016491) and transferase activity (GO:0016740) (Figure 1A). Notably, one of the most significant GO predictions included antioxidant activity (GO:0016209). The most significant molecular pathways involving a larger number of genes include the detoxification of ROS, cellular responses to chemical stress, glutathione conjugation, phase II-conjugation of compounds, biological oxidations, and cellular responses to stress (Figure 1B). Remarkably, three genes play a key role in the FOXO-mediated transcription pathway (Figure 1B), underscoring their specific role in these cellular processes.

### 3.2. Expression and Intraovarian Localization of the Antioxidant Enzymes

Computational analysis by the Ovarian Kaleidoscope database revealed that all the selected antioxidant enzymes are expressed in the human ovary at different levels, and 15 mRNAs were found inside the exosomes (Table 1).

Concerning their intraovarian localization, GCLC, GCLM, GLRX2, GSR, SOD1, SOD2, and TXNRD1 were found in the oocyte. Only SOD1 and SOD2 showed ubiquitous intraovarian expression. The localization of TXRND2, PRDX3, MGST1, GSPT1, and GSTM1 remained undetermined. Unique localization was shown by GPX1, reported in LCs; GSTA4, present in the T compartment; GSTT1 and TNX2 in the GCs; and TXNRD1 in the oocyte (Table 2).

**Figure 2:**
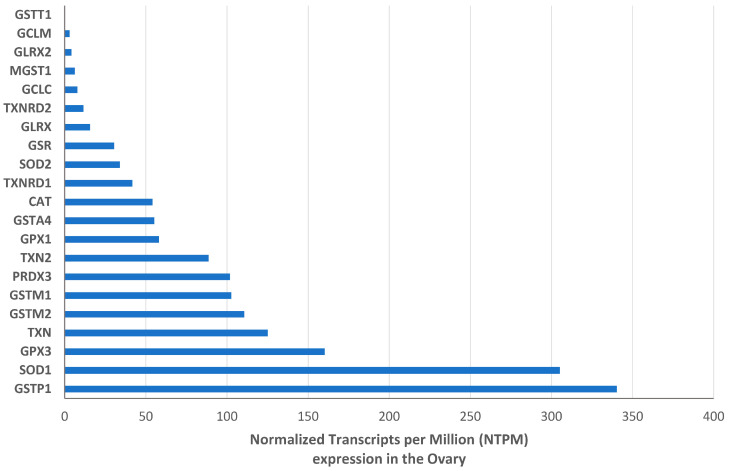
Expression of the selected antioxidant enzymes, in terms of transcripts, within the ovarian tissue.

Transcriptome data analysis revealed a higher expression level of GSTP1, SOD1, and GPX3, with 340.3, 305.2, and 160.3 normalized transcripts per million (NTPM) in the human ovary, respectively (Figure 2).

### 3.3. LncRNA-miRNA-mRNA Competing Endogenous RNA Networks

The search for miRNAs targeting the antioxidant enzyme mRNAs produced results for some of the initially selected enzymes. Specifically, we found 10 mRNAs targeted by 22 miRNAs (Table 3). 

**Figure 3:**
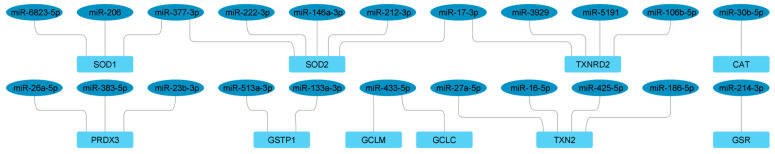
Network showing the interactions between miRNAs and antioxidant enzyme mRNAs.

For the protein-encoding genes not listed in the table, no data on miRNAs targeting them are currently available in public databases or the literature. As shown in Table 3 and Figure 3, different target mRNAs may be regulated by the same miRNAs, and multiple miRNAs may regulate a single mRNA (Figure 3).

The search for the lncRNAs with at least two target binding sites for the 22 miRNAs returned only 10 miRNAs sponged by the 22 lncRNAs (Table 4).

**Figure 4:**
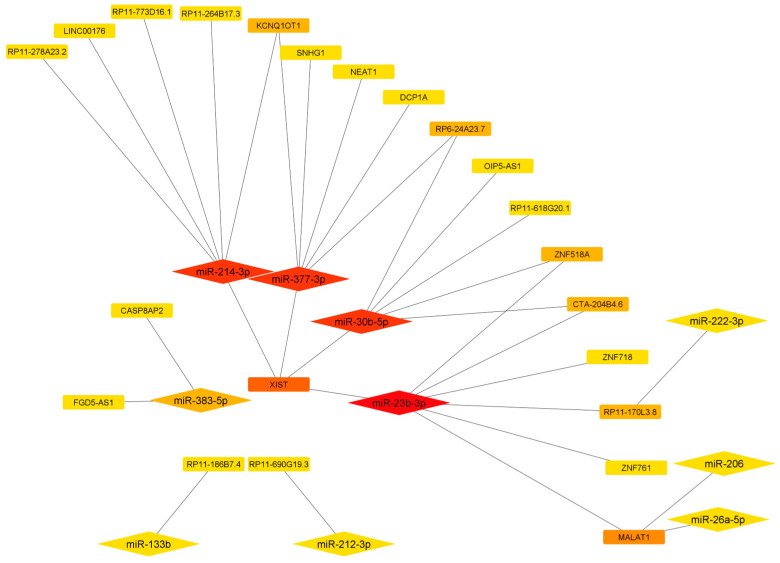
Network showing the interaction between the miRNAs targeting antioxidant enzymes and lncRNAs. The nodes are ranked according to the degree scoring method, with a color scheme from highly central (red) to central (yellow).

As reported in Figure 4, a single lncRNA can sponge different miRNAs, and a single miRNA can interact with different lncRNAs. Among the identified lncRNAs, XIST and MALAT1 show the highest number of interactors (Table 4 and Figure 4).

**Figure 5:**
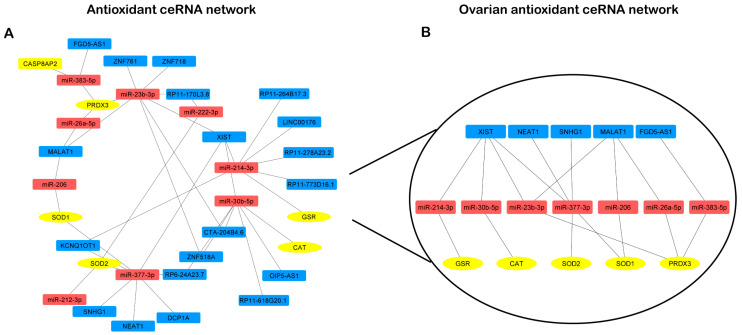
mRNA-miRNA-lncRNA ceRNA network. (**A**) Antioxidant ceRNA network. (**B**) Ovarian antioxidant OvAnOx ceRNA network. miRNAs are red-colored, mRNAs are yellow-colored, and lncRNAs are blue-colored.

As reported in Section 2, the competing endogenous RNA networks (ceRNA network) were designed considering the miRNAs targeting antioxidant enzymes mRNAs and the lncRNAs targeting these miRNAs. The resulting ceRNA network, named the “antioxidant ceRNA network”, showed that PRDX3, SOD1, SOD2, GSR, and CAT transcripts can take part in different regulatory loops involving lncRNAs and miRNAs (Figure 5A)

To identify the ovarian “antioxidant ceRNA network”, we focused on lncRNAs and miRNAs expressed in the ovary. We found 10 miRNAs and 5 lncRNAs interacting with our 5 mRNAs inside the ovary. The network depicted in Figure 5B represents the identified lncRNAs that are part of different redundant networks. Through the regulation of four miRNAs, XIST may control GSR, CAT, SOD2, SOD1, and PRDX3. By sponging three miRNAs, MALAT1 may control three of them: SOD2, SOD1, and PRDX3. The action of NEAT and SNHG1 seems to specifically target the superoxide activity, whereas FGD5-AS1 is involved only in PRDX3 regulation (Figure 5B).

## 4. Discussion

The ovarian function relies on a fine regulation of redox balance, which governs follicular development by activating specific pathways and preventing oxidative damage to germ cells. ROS signaling is a double-edged sword, playing essential roles in normal ovarian function and contributing to various ovarian pathologies when dysregulated [2,4,5]. During follicular development, moderate levels of ROS act as signaling molecules crucial for follicular maturation, oocyte meiosis, and ovulation. Controlled ROS levels ensure the atresia of non-dominant follicles, allowing only the healthiest to mature. The LH surge increases ROS production, facilitating follicular wall breakdown and oocyte release [23]. ROS also play a key role in the inflammatory response essential for ovulation and regulate genes involved in proteolysis and tissue remodeling [23,24]. Additionally, ROS impact luteal cell survival and function, affecting progesterone production, luteal phase duration, and angiogenesis for corpus luteum maintenance [6,25,26,27]. Accumulating evidence demonstrates that ROS are key signals in the initiation of apoptosis in antral follicles and granulosa cells of antral follicles by diverse stimuli, such as gonadotropin withdrawal, exposure to exogenous toxicants, and exposure to ionizing radiation, and that antioxidants protect against these stimuli [28].

In the present study, by focusing on mRNAs and ncRNAs present in the ovary and taking into account only validated ncRNA interactions, we built an ovarian antioxidant ceRNA network, named OvAnOx ceRNA, comprising 5 mRNAs (SOD1, SOD2, CAT, PRDX3, GR), 10 miRNAs, and 5 lncRNAs (XIST, FGD5-AS1, MALAT1, NEAT1, SNHG1). Following a discussion of the results regarding the antioxidant enzymes studied, the main components of OvAnOx ceRNA will be discussed with reference to their role in the regulation of ovarian antioxidant activity and cellular processes.

### 4.1. Antioxidant Genes in the Human Ovary

According to the results, the genes included in our analysis are representative of all the catalytic reactions involved in ROS detoxification in the human ovary. When we focused on functional pathways, it emerged that our genes of interest are involved in the cellular response to stress conditions, detoxification of ROS, biological oxidation, phase-II detoxification, GSH conjugation, and FOXO-mediated transcription. FOXO transcription factors work together with Nrf2 to upregulate the expression of antioxidant enzymes, providing a coordinated defense against oxidative stress [29,30]. In accordance with our bioinformatics analysis, the 21 enzymes under study cover antioxidant activities in different intracellular and extracellular compartments. Indeed, most of them have been described as exosome cargo. A peculiar distribution in the oocyte, granulosa and theca cells, and the extracellular environment is also reported. Notably, only one paper described the presence of antioxidant enzymes in exosomes released in the culture media of mammalian granulosa cells [31]. The observation that TXNRD1 is uniquely expressed in the oocyte, GSTT1 and TXN2 in GCs, and GPX1 and GSTM2 in LCs, might deserve attention in an attempt to characterize the role of antioxidant enzymes in the ovary.

Among the selected enzymes, the most expressed gene is GSTP1, followed by SOD1 and GPX3, suggesting a prominent role of these genes in the ovarian antioxidant defense.

SODs are involved in the initial and most important step for controlling the redox state by catalyzing the transformation of anion superoxide (O2^•-^) into molecular oxygen (O_2_) and hydrogen peroxide (H_2_O_2_) [32]. Superoxide anion is one of the first ROS formed during the reduction of molecular oxygen during metabolism and plays a key role in redox signaling pathways. Catalase (CAT), PRDX (peroxiredoxin), and GPX catalyze the conversion of hydrogen peroxide into water after the dismutation event [33,34,35,36]. In addition to GPX, many enzymes included in this study use glutathione (GSH) as an electron donor. Many reductive cellular enzyme systems depend upon the use of the tripeptide glutathione. Reduced GSH is oxidized to GSSG (oxidized glutathione) by GPX. The conversion of GSSG to GSH via glutathione reductase (GSR) with NADPH consumption is a common enzymatic method for sustaining GSH in most tissues [37,38]. Thus, the ability of cells to scavenge oxidants is fundamentally dependent on this entire process, known as “GSH recycling” [39]. By catalyzing the conjugation of reactive metabolites with GSH, glutathione transferase (GST) is essential in the detoxification process [40]. Glutamate cysteine ligase (GCL) and glutathione synthetase (GS) can catalyze the de novo synthesis of GSH from glutamate, cysteine, and glycine [41]. Glutaredoxin (GRX) (also known as thioltransferase) catalyzes the reduction of protein disulfides and mixed disulfides between proteins and GSH [42]. An important reductive system is represented by thioredoxin reductase (TXNRD) and thioredoxin (TRX) [43,44]. TRX reduces oxidized proteins by donating electrons, which are replenished by TRXRD using NADPH [43,44].

### 4.2. The OvAnOx ceRNA Network

#### 4.2.1. mRNA Components

The mRNA components of the OvAnOx ceRNA network, SOD1, SOD2, CAT, GSR, and PRDX3, form a critical network for defense against oxidative stress and maintenance of a redox state suitable for proper ovarian function. Numerous knockout mouse models have been used to explore the role of the enzymes included in the OvAnOx ceRNA network. SOD1-deficient mice show reduced fertility, with a reduction in preovulatory follicles and corpora lutea [45]. By contrast, in SOD2-deficient mice, all follicular phases were detected, and viable pups were produced when their ovaries were transplanted into wild-type mice, indicating that SOD2 plays a less significant role than SOD1 [45]. There were no changes in the fertility of mice with an inactivating mutation in the GSR gene [46,47] or in the CAT gene [48].

SOD1 and SOD2 are absent in primordial and primary follicles. SOD2 appears in secondary follicles, while SOD1 is first seen in theca cells after antral cavity formation and in granulosa cells at the dominant follicle stage [49]. Both isoforms are found in follicular fluid, with increased amounts and activity during antral development [49]. In luteinized granulosa and theca cells, SOD1 and SOD2 are highly expressed. Their enzymatic activity decreases with follicular growth, potentially inhibiting estrogen synthesis by suppressing FSH-induced aromatase in granulosa cells. SOD activity peaks at proestrus with reduced superoxide radicals compared to the estrous stage [49]). During corpus luteum regression, increased ROS levels coincide with reduced SOD1 and increased SOD2, addressing mitochondrial ROS from cytokines and inflammation [50]. Aging is linked to decreased SODs and catalase in granulosa cells, contributing to reproductive decline [51]. Oxidative stress from SOD2 deficiency inhibits progestin and estradiol production in granulosa cells by affecting key steroidogenic enzymes, and SOD1 activity varies in women with PCOS [52,53].

Oocytes experience increased ROS levels due to active metabolism in the preovulatory follicle and ovulation [23,54]. They express all three SOD isoforms, with SOD1 and SOD3 in the nucleus, protecting DNA and regulating redox-sensitive gene transcription [55,56]. Age-related oxidative damage causes meiotic segregation errors, mitigated by extra SOD1 or SOD2 [57]. Oocytes have lower catalase expression compared to other cells, but catalase protects DNA during meiotic maturation and is involved in follicle development, the estrous cycle, and ovarian steroidogenesis [56]. Catalase activity increases in granulosa cells during ovarian growth and luteinization, aiding follicle selection and preventing ROS-mediated apoptosis in dominant follicles [51,58,59].

GSH synthesized in oocytes regulates the sulfur–oxygen reduction state, promotes cytoplasmic maturation, and protects against oxidative stress, improving spindle function and embryo development [60,61]. GSR expression decreases in aging oocytes, leading to oxidative damage and ovarian decline [62,63], but is highest during metestrus, which is crucial for reproduction. GSH is essential for oocyte competence, influenced by gonadotropin signaling [64]. Oocytes have the highest GSR activity in the ovary, with GSH levels in cumulus cells increasing during maturation [65]. FSH therapy promotes GSH synthesis and prevents apoptosis in antral follicles, but its antiapoptotic effect is reduced if GSH synthesis is inhibited [66,67].

PRDX3 expression decreases during the luteinization of preovulatory follicles in pigs and is stimulated by gonadotropins in theca cells, aiding the antioxidant system during ovulation. In aged mouse oocytes, Prdx3 mRNA expression is reduced, increasing oxidative stress sensitivity [68]. Mitochondrial antioxidants Prdx3 decrease with age in mouse ovaries [22].

#### 4.2.2. lncRNAs Components

In recent years, the role of lncRNA in oxidative stress has emerged, specifically in oxidative stress-related diseases such as neurodegenerative pathologies, atherosclerosis, and diabetes [69,70]. There has been limited progress in understanding the role of ceRNAs in female reproductive diseases, particularly in PCOS [17,71,72,73,74,75,76,77,78,79,80]), indicating that the effect of ceRNAs in female reproduction is poorly understood and needs to be further explored. To the best of our knowledge, no studies have investigated their role in the regulation of ovarian OS, a condition known to be involved in female reproductive dysfunctions [2,4,5,6].

The lncRNA XIST triggers X chromosome inactivation [81] and regulates oocyte loss by suppressing miR-23b-3p/miR-29a-3p and upregulating STX17 in perinatal mouse [2] ovaries [82]. Highly expressed in fetal ovaries, XIST is downregulated after birth as the primordial follicle pool forms. XIST accelerates oocyte autophagy during perinatal oocyte loss [82]. XIST is expressed early in unfertilized oocytes and pronuclei-stage zygotes [83]. A ceRNA network incorporating XIST was constructed to predict differences in GCs from patients with EM [84]. XIST is downregulated in the serum of PCOS patients and is correlated with adverse pregnancy outcomes [85].

MALAT1 influences the oxidative stress response, acting as an antioxidant by lowering Keap1 levels, thereby activating and stabilizing Nrf2 in H_2_O_2_-induced human umbilical vein endothelial cells (HUVECs). This enhances antioxidant capacity and reduces oxidative damage. MALAT1 also regulates Nrf2 and, in addition, can activate the p38MAPK pathway to modulate apoptosis and oxidative stress [86,87]. In ovarian function, MALAT1 knockdown increases apoptosis and reduces proliferation in granulosa cells by promoting P53 degradation [88]. MALAT1 regulates ovarian follicular atresia, apoptosis, and steroid synthesis, and is upregulated in KGN cells after AMH stimulation [89,90]. PCOS patients show lower MALAT1 levels, suggesting its potential role in PCOS pathogenesis and targeted therapy [73].

NEAT1, a highly conserved lncRNA, is highly expressed in PCOS patients, promoting the expression of androgen receptor (AR), follistatin (FST), and IRS-2, which are potentially involved in PCOS pathogenesis [73]. NEAT1 exacerbates metabolic disorders in PCOS mice by downregulating miR-324-3p and upregulating BRD3 [91]. In Neat1 knockout mice, corpus luteum formation is impaired, leading to decreased fertility, which can be partially rescued by progesterone [92]. NEAT1 is downregulated in premature ovarian failure (POF) mice, where it modulates the STC2/MAPK pathway to reduce apoptosis and autophagy [93].

#### 4.2.3. The miRNAs Components

Over the last decade, research has highlighted the regulatory interplay between miRNAs and redox signaling. Oxidative stress can regulate miRNAs, and miRNAs can influence cellular redox status [94]. ROS exposure can inhibit Dicer activity, delaying miRNA maturation, and can also affect pri-miRNA structures and promoter methylation [95]. Many ROS-responsive miRNAs, in turn, influence the Nrf2 system [69,95,96].

Specific miRNAs play crucial roles in ovarian function and oxidative stress regulation [97,98,99,100,101,102]. miR-214 offers protection against oxidative damage by targeting GSR and cytochrome P450 oxidoreductase (POR) and is involved in cell survival, embryonic development, and ovarian cancer resistance [103]. miR-23b-3p promotes oocyte autophagy by reducing mature miR-23b-3p levels, which is crucial for oocyte death regulation [82]. miR-377-3p is proposed as a marker of oocyte quality, aiding in predicting ovarian superovulation potential [104]. miR-206 is linked to PCOS, regulates granulosa cell viability and apoptosis via the PI3K/AKT pathway, and is a potential biomarker for superovulation response [105,106,107,108].

Additionally, miR-206 regulates oocyte maturation and granulosa cell development by targeting AURKA [105,109]. RNAseq analysis in goat ovary showed miR-206 upregulation in ovarian stroma, indicating roles in ovarian organogenesis and hormone secretion by oocyte meiosis [109].

miR-26a-5p is upregulated in PCOS, involved in corpus luteum development, and plays a key role in reproductive span regulation. miR-383-5p decreases in PCOS patients, suppresses the PI3K/AKT pathway, and enhances KGN cell apoptosis [110,111,112,113,114]. These miRNAs modulate redox status and are crucial for ovarian health, influencing processes from oocyte maturation to hormone secretion and disease resistance [115].

### 4.3. Clinical Implications

Many reproductive disorders, such as polycystic ovarian syndrome (PCOS), endometriosis, and unexplained infertility, are pathological effects of decreased antioxidant defense systems. Decreased antioxidant systems have also been linked to age-related declines in reproductive function. Considering the importance of redox balance in ovarian functions and the ongoing debate on the efficacy of antioxidant therapies in the treatment of female fertility [7], the results of this bioinformatic study represent a valuable contribution to the knowledge of selectively targeting redox-modulating systems in reproductive medicine. Experimental validation of alterations in the OvAnOx ceRNA network in ovarian disorders would contribute to exploring innovative biomarkers and potential drug molecules based on components of this network.

## 5. Conclusions

In conclusion, our findings, supported by the literature, indicate that all components of the OvAnOx ceRNA network play significant roles in ovarian physiology. Through our bioinformatic analysis, we identified that antioxidant activity, particularly involving superoxide and hydrogen peroxide scavenging and glutathione recycling, is regulated by ncRNAs, which are also implicated in various ovarian functions beyond redox modulation. We predict that antioxidant enzymes (e.g., SOD1, SOD2, CAT, GRS, and PRDX3) function within a complex regulatory network that integrates signals from multiple intracellular processes, including the regulation of ovarian reserve, follicular dynamics, apoptosis, and oocyte maturation under both physiological and pathological conditions. These findings suggest that the OvAnOx ceRNA network could be a valuable tool for exploring the intricate interplay between redox potential and ovarian signaling pathways, with implications for reproductive health, aging, and disease.

## Figures and Tables

**Table 1:** Localization of the 21 antioxidant enzymes.

	Cellular Localization		Extracellular Localization
Gene Name	Cytoplasm	Mitochondria	Exosomes
CAT			
GCLC			
GCLM			
GLRX			
GLRX2			
GPX1			
GPX3			
GSR			
GSTA4			
GSTM1			
GSTM2			
GSTP1			
GSTT1			
MGST1			
PRDX3			
SOD1			
SOD2			
TXN			
TXN2			
TXNRD1			
TXNRD2			

The grey color indicates the presence of antioxidant enzymes. Abbreviations: CAT: catalase; GCLC: glutamate–cysteine ligase catalytic subunit; GCLM: glutamate–cysteine ligase modifier subunit; GLRX: glutaredoxin; GLRX2: glutaredoxin 2; GPX1: glutathione peroxidase 1; GPX3: glutathione peroxidase 3; GSR: glutathione reductase; GSTA4: glutathione s-transferase alpha 4; GSTM1: glutathione s-transferase Mu 1; GSTM2: glutathione s-transferase Mu 2; GSTP1: glutathione s-transferase Pi 1; GSTT1: glutathione s-transferase theta 1; MGST1: microsomal glutathione s-transferase 1; PRDX3: peroxiredoxin 3; SOD1: superoxide dismutase 1; SOD2: superoxide dismutase 2; TXN: thioredoxin; TXN2: thioredoxin 2; TXNRD1: thioredoxin reductase 1; TXNRD2: thioredoxin reductase 2.

**Table 2:** Ovarian localization of the 21 antioxidant enzymes.

Gene Name	FF	O	CC	GC	TC	LC	SC	ND
CAT								
GCLC								
GCLM								
GLRX								
GLRX2								
GPX1								
GPX3								
GSR								
GSTA4								
GSTM1								
GSTM2								
GSTP1								
GSTT1								
MGST1								
PRDX3								
SOD1								
SOD2								
TXN								
TXN2								
TXNRD1								
TXNRD2								

The grey color indicates the presence of antioxidant enzymes. Abbreviations: Follicular fluid (FF); oocyte (O); cumulus cells (CC); granulosa cells (GC); theca cells (TC); luteal cells (LC); stromal cells (SC); Not Determined (ND).

**Table 3:** miRNAs regulating the 21 antioxidant enzyme mRNAs.

	CAT	GCLC	GCLM	GSR	GSTP1	PRDX3	SOD1	SOD2	TXN2	TXNRD2
miR-16-5p										
miR-17-3p										
miR-23b-3p										
miR-26a-5p										
miR-27a-5p										
miR-30b-5p										
miR-106b										
miR-133a										
mir-146a										
miR-186-5p										
miR-206										
miR-212-3p										
miR-214-3p										
miR-222-3p										
miR-377-3p										
miR-383-3p										
miR-425-5p										
miR-433-5p										
miR-513a-3p										
miR-3929										
miR-5191										
miR-6823-5p										

The grey color indicates predicted miRNA-mRNA interactions.

**Table 4:** miRNAs regulating 21 antioxidant enzyme mRNAs are sponged by lncRNAs.

	miR-23b-3p	miR-26a-5p	miR-30b-5p	miR-133b	miR-206	miR-212-3p	miR-214-3p	miR-222-3p	miR-377-3p	miR-383-5p
CASP8AP2										
CTA-204B4.6										
DCP1A										
FGD5-AS1										
KCNQ1OT1										
LINC00176										
MALAT1										
NEAT1										
OIP5-AS1										
RP11-170L3.8										
RP11-186B7.4										
RP11-264B17.3										
RP11-278A23.2										
RP11-618G20.1										
RP11-690G19.3										
RP11-773D16.1										
RP6-24A23.7										
SNHG1										
XIST										
ZNF518A										
ZNF718										
ZNF761										

The grey color indicates predicted miRNA-lncRNA interactions.

## Data Availability

The data presented in this study are available on request from the corresponding author.

## References

[1] Gershon E., Dekel N. (2020). Newly Identified Regulators of Ovarian Folliculogenesis and Ovulation. Int. J. Mol. Sci..

[2] Tatone C., Amicarelli F., Carbone M.C., Monteleone P., Caserta D., Marci R., Artini P.G., Piomboni P., Focarelli R. (2008). Cellular and molecular aspects of ovarian follicle ageing. Hum. Reprod. Update.

[3] Tatone C., Di Emidio G., Placidi M., Rossi G., Ruggieri S., Taccaliti C., D’Alfonso A., Amicarelli F., Guido M. (2021). AGEs-related dysfunctions in PCOS: Evidence from animal and clinical research. J. Endocrinol..

[4] Ruder E.H., Hartman T.J., Goldman M.B. (2009). Impact of oxidative stress on female fertility. Curr. Opin. Obstet. Gynecol..

[5] Yan F., Zhao Q., Li Y., Zheng Z., Kong X., Shu C., Liu Y., Shi Y. (2022). The role of oxidative stress in ovarian aging: A review. J. Ovarian. Res..

[6] Liang J., Gao Y., Feng Z., Zhang B., Na Z., Li D. (2023). Reactive oxygen species and ovarian diseases: Antioxidant strategies. Redox Biol..

[7] Showell M.G., Mackenzie-Proctor R., Jordan V., Hart R.J. (2020). Antioxidants for female subfertility. Cochrane Database Syst. Rev..

[8] de Almeida A., de Oliveira J., da Silva Pontes L.V., de Souza Junior J.F., Goncalves T.A.F., Dantas S.H., de Almeida Feitosa M.S., Silva A.O., de Medeiros I.A. (2022). ROS: Basic Concepts, Sources, Cellular Signaling, and its Implications in Aging Pathways. Oxid. Med. Cell. Longev..

[9] Sies H., Mailloux R.J., Jakob U. (2024). Fundamentals of redox regulation in biology. Nat. Rev. Mol. Cell Biol..

[10] Lei X.G., Zhu J.H., Cheng W.H., Bao Y., Ho Y.S., Reddi A.R., Holmgren A., Arner E.S. (2016). Paradoxical Roles of Antioxidant Enzymes: Basic Mechanisms and Health Implications. Physiol. Rev..

[11] Nemeth K., Bayraktar R., Ferracin M., Calin G.A. (2024). Non-coding RNAs in disease: From mechanisms to therapeutics. Nat. Rev. Genet..

[12] Mattick J.S., Amaral P.P., Carninci P., Carpenter S., Chang H.Y., Chen L.L., Chen R., Dean C., Dinger M.E., Fitzgerald K.A. (2023). Long non-coding RNAs: Definitions, functions, challenges and recommendations. Nat. Rev. Mol. Cell Biol..

[13] Gou L.T., Zhu Q., Liu M.F. (2023). Small RNAs: An expanding world with therapeutic promises. Fundam. Res..

[14] Zhang P., Wu W., Chen Q., Chen M. (2019). Non-Coding RNAs and their Integrated Networks. J. Integr. Bioinform..

[15] Tay Y., Rinn J., Pandolfi P.P. (2014). The multilayered complexity of ceRNA crosstalk and competition. Nature.

[16] Caponnetto A., Ferrara C., Fazzio A., Agosta N., Scribano M., Vento M.E., Borzì P., Barbagallo C., Stella M., Ragusa M. (2024). A Circular RNA Derived from the Pumilio 1 Gene Could Regulate PTEN in Human Cumulus Cells. Genes.

[17] Caponnetto A., Battaglia R., Ferrara C., Vento M.E., Borzì P., Paradiso M., Scollo P., Purrello M., Longobardi S., D’Hooghe T. (2022). Down-regulation of long non-coding RNAs in reproductive aging and analysis of the lncRNA-miRNA-mRNA networks in human cumulus cells. J. Assist. Reprod. Genet..

[18] Barbagallo D., Palermo C.I., Barbagallo C., Battaglia R., Caponnetto A., Spina V., Ragusa M., Di Pietro C., Scalia G., Purrello M. (2022). Competing endogenous RNA network mediated by circ_3205 in SARS-CoV-2 infected cells. Cell Mol. Life Sci..

[19] Nejadi Orang F., Abdoli Shadbad M. (2024). Competing endogenous RNA networks and ferroptosis in cancer: Novel therapeutic targets. Cell Death Dis..

[20] Ciesielska S., Slezak-Prochazka I., Bil P., Rzeszowska-Wolny J. (2021). Micro RNAs in Regulation of Cellular Redox Homeostasis. Int. J. Mol. Sci..

[21] Kinoshita C., Aoyama K. (2021). The Role of Non-Coding RNAs in the Neuroprotective Effects of Glutathione. Int. J. Mol. Sci..

[22] Lim J., Luderer U. (2011). Oxidative damage increases and antioxidant gene expression decreases with aging in the mouse ovary. Biol. Reprod..

[23] Shkolnik K., Tadmor A., Ben-Dor S., Nevo N., Galiani D., Dekel N. (2011). Reactive oxygen species are indispensable in ovulation. Proc. Natl. Acad. Sci. USA.

[24] Kala M., Shaikh M.V., Nivsarkar M. (2017). Equilibrium between anti-oxidants and reactive oxygen species: A requisite for oocyte development and maturation. Reprod. Med. Biol..

[25] Jamil M., Debbarh H., Aboulmaouahib S., Aniq Filali O., Mounaji K., Zarqaoui M., Saadani B., Louanjli N., Cadi R. (2020). Reactive oxygen species in reproduction: Harmful, essential or both?. Zygote.

[26] Tian Y., Liu X., Pei X., Gao H., Pan P., Yang Y. (2022). Mechanism of Mitochondrial Homeostasis Controlling Ovarian Physiology. Endocrinology.

[27] Wang L., Tang J., Wang L., Tan F., Song H., Zhou J., Li F. (2021). Oxidative stress in oocyte aging and female reproduction. J. Cell. Physiol..

[28] Devine P.J., Perreault S.D., Luderer U. (2012). Roles of reactive oxygen species and antioxidants in ovarian toxicity. Biol. Reprod..

[29] Klotz L.O., Sanchez-Ramos C., Prieto-Arroyo I., Urbanek P., Steinbrenner H., Monsalve M. (2015). Redox regulation of FoxO transcription factors. Redox Biol..

[30] Tonelli C., Chio I.I.C., Tuveson D.A. (2018). Transcriptional Regulation by Nrf2. Antioxid. Redox Signal..

[31] Saeed-Zidane M., Linden L., Salilew-Wondim D., Held E., Neuhoff C., Tholen E., Hoelker M., Schellander K., Tesfaye D. (2017). Cellular and exosome mediated molecular defense mechanism in bovine granulosa cells exposed to oxidative stress. PLoS ONE.

[32] Case A.J. (2017). On the Origin of Superoxide Dismutase: An Evolutionary Perspective of Superoxide-Mediated Redox Signaling. Antioxidants.

[33] Cao Z., Bhella D., Lindsay J.G. (2007). Reconstitution of the mitochondrial PrxIII antioxidant defence pathway: General properties and factors affecting PrxIII activity and oligomeric state. J. Mol. Biol..

[34] Yewdall N.A., Peskin A.V., Hampton M.B., Goldstone D.C., Pearce F.G., Gerrard J.A. (2018). Quaternary structure influences the peroxidase activity of peroxiredoxin 3. Biochem. Biophys. Res. Commun..

[35] Anwar S., Alrumaihi F., Sarwar T., Babiker A.Y., Khan A.A., Prabhu S.V., Rahmani A.H. (2024). Exploring Therapeutic Potential of Catalase: Strategies in Disease Prevention and Management. Biomolecules.

[36] Trenz T.S., Delaix C.L., Turchetto-Zolet A.C., Zamocky M., Lazzarotto F., Margis-Pinheiro M. (2021). Going Forward and Back: The Complex Evolutionary History of the GPx. Biology.

[37] Chen T.H., Wang H.C., Chang C.J., Lee S.Y. (2024). Mitochondrial Glutathione in Cellular Redox Homeostasis and Disease Manifestation. Int. J. Mol. Sci..

[38] Couto N., Wood J., Barber J. (2016). The role of glutathione reductase and related enzymes on cellular redox homoeostasis network. Free Radic. Biol. Med..

[39] Hayes J.D., McLellan L.I. (1999). Glutathione and glutathione-dependent enzymes represent a co-ordinately regulated defence against oxidative stress. Free Radic. Res..

[40] Mazari A.M.A., Zhang L., Ye Z.W., Zhang J., Tew K.D., Townsend D.M. (2023). The Multifaceted Role of Glutathione S-Transferases in Health and Disease. Biomolecules.

[41] Zhang H., Forman H.J. (2012). Glutathione synthesis and its role in redox signaling. Semin. Cell. Dev. Biol..

[42] Allen E.M., Mieyal J.J. (2012). Protein-thiol oxidation and cell death: Regulatory role of glutaredoxins. Antioxid. Redox Signal..

[43] Song Z., Fan C., Zhao J., Wang L., Duan D., Shen T., Li X. (2023). Fluorescent Probes for Mammalian Thioredoxin Reductase: Mechanistic Analysis, Construction Strategies, and Future Perspectives. Biosensors.

[44] Yang B., Lin Y., Huang Y., Shen Y.Q., Chen Q. (2024). Thioredoxin (Trx): A redox target and modulator of cellular senescence and aging-related diseases. Redox Biol..

[45] Matzuk M.M., Dionne L., Guo Q., Kumar T.R., Lebovitz R.M. (1998). Ovarian function in superoxide dismutase 1 and 2 knockout mice. Endocrinology.

[46] Pretsch W. (1999). Glutathione reductase activity deficiency in homozygous Gr1a1Neu mice does not cause haemolytic anaemia. Genet. Res..

[47] Rogers L.K., Bates C.M., Welty S.E., Smith C.V. (2006). Diquat induces renal proximal tubule injury in glutathione reductase-deficient mice. Toxicol. Appl. Pharmacol..

[48] Ho Y.S., Xiong Y., Ma W., Spector A., Ho D.S. (2004). Mice lacking catalase develop normally but show differential sensitivity to oxidant tissue injury. J. Biol. Chem..

[49] Wang S., He G., Chen M., Zuo T., Xu W., Liu X. (2017). The Role of Antioxidant Enzymes in the Ovaries. Oxid. Med. Cell. Longev..

[50] Vu H.V., Lee S., Acosta T.J., Yoshioka S., Abe H., Okuda K. (2012). Roles of prostaglandin F2alpha and hydrogen peroxide in the regulation of Copper/Zinc superoxide dismutase in bovine corpus luteum and luteal endothelial cells. Reprod. Biol. Endocrinol..

[51] Tatone C., Carbone M.C., Falone S., Aimola P., Giardinelli A., Caserta D., Marci R., Pandolfi A., Ragnelli A.M., Amicarelli F. (2006). Age-dependent changes in the expression of superoxide dismutases and catalase are associated with ultrastructural modifications in human granulosa cells. Mol. Hum. Reprod..

[52] Bizon A., Tchorz A., Madej P., Lesniewski M., Wojtowicz M., Piwowar A., Franik G. (2022). The Activity of Superoxide Dismutase, Its Relationship with the Concentration of Zinc and Copper and the Prevalence of rs2070424 Superoxide Dismutase Gene in Women with Polycystic Ovary Syndrome-Preliminary Study. J. Clin. Med..

[53] Seleem A.K., El Refaeey A.A., Shaalan D., Sherbiny Y., Badawy A. (2014). Superoxide dismutase in polycystic ovary syndrome patients undergoing intracytoplasmic sperm injection. J. Assist. Reprod. Genet..

[54] Pandey A.N., Chaube S.K. (2014). A moderate increase of hydrogen peroxide level is beneficial for spontaneous resumption of meiosis from diplotene arrest in rat oocytes cultured in vitro. Biores. Open Access.

[55] Matos L., Stevenson D., Gomes F., Silva-Carvalho J.L., Almeida H. (2009). Superoxide dismutase expression in human cumulus oophorus cells. Mol. Hum. Reprod..

[56] Cetica P.D., Pintos L.N., Dalvit G.C., Beconi M.T. (2001). Antioxidant enzyme activity and oxidative stress in bovine oocyte in vitro maturation. IUBMB Life.

[57] Perkins A.T., Greig M.M., Sontakke A.A., Peloquin A.S., McPeek M.A., Bickel S.E. (2019). Increased levels of superoxide dismutase suppress meiotic segregation errors in aging oocytes. Chromosoma.

[58] Behl R., Pandey R.S. (2002). FSH induced stimulation of catalase activity in goat granulosa cells in vitro. Anim. Reprod. Sci..

[59] Serke H., Bausenwein J., Hirrlinger J., Nowicki M., Vilser C., Jogschies P., Hmeidan F.A., Blumenauer V., Spanel-Borowski K. (2010). Granulosa cell subtypes vary in response to oxidized low-density lipoprotein as regards specific lipoprotein receptors and antioxidant enzyme activity. J. Clin. Endocrinol. Metab..

[60] Barros F.D.A., Adona P.R., Guemra S., Damiao B.C.M. (2019). Oxidative homeostasis in oocyte competence for in vitro embryo development. Anim. Sci. J..

[61] Luciano A.M., Goudet G., Perazzoli F., Lahuec C., Gerard N. (2006). Glutathione content and glutathione peroxidase expression in in vivo and in vitro matured equine oocytes. Mol. Reprod. Dev..

[62] Katz-Jaffe M.G., Lane S.L., Parks J.C., McCallie B.R., Makloski R., Schoolcraft W.B. (2020). Antioxidant Intervention Attenuates Aging-Related Changes in the Murine Ovary and Oocyte. Life.

[63] Wang S., Zheng Y., Li J., Yu Y., Zhang W., Song M., Liu Z., Min Z., Hu H., Jing Y. (2020). Single-Cell Transcriptomic Atlas of Primate Ovarian Aging. Cell.

[64] Dumollard R., Ward Z., Carroll J., Duchen M.R. (2007). Regulation of redox metabolism in the mouse oocyte and embryo. Development.

[65] Kaneko T., Iuchi Y., Kawachiya S., Fujii T., Saito H., Kurachi H., Fujii J. (2001). Alteration of glutathione reductase expression in the female reproductive organs during the estrous cycle. Biol. Reprod..

[66] Tsai-Turton M., Luderer U. (2006). Opposing effects of glutathione depletion and follicle-stimulating hormone on reactive oxygen species and apoptosis in cultured preovulatory rat follicles. Endocrinology.

[67] Hoang Y.D., Nakamura B.N., Luderer U. (2009). Follicle-stimulating hormone and estradiol interact to stimulate glutathione synthesis in rat ovarian follicles and granulosa cells. Biol. Reprod..

[68] Park J.I., Jeon H.J., Jung N.K., Jang Y.J., Kim J.S., Seo Y.W., Jeong M., Chae H.Z., Chun S.Y. (2012). Periovulatory expression of hydrogen peroxide-induced sulfiredoxin and peroxiredoxin 2 in the rat ovary: Gonadotropin regulation and potential modification. Endocrinology.

[69] Hernández-Cruz E.Y., Arancibia-Hernández Y.L., Loyola-Mondragón D.Y., Pedraza-Chaverri J. (2022). Oxidative Stress and Its Role in Cd-Induced Epigenetic Modifications: Use of Antioxidants as a Possible Preventive Strategy. Oxygen.

[70] Infante-Menendez J., Gonzalez-Lopez P., Huertas-Larez R., Gomez-Hernandez A., Escribano O. (2023). Oxidative Stress Modulation by ncRNAs and Their Emerging Role as Therapeutic Targets in Atherosclerosis and Non-Alcoholic Fatty Liver Disease. Antioxidants.

[71] Fan H., Zhou D., Zhang X., Jiang M., Kong X., Xue T., Gao L., Lu D., Tao C., Wang L. (2023). hsa_circRNA_BECN1 acts as a ceRNA to promote polycystic ovary syndrome progression by sponging the miR-619-5p/Rab5b axis. Mol. Hum. Reprod..

[72] Bai L., Gong J., Guo Y., Li Y., Huang H., Liu X. (2022). Construction of a ceRNA network in polycystic ovary syndrome (PCOS) driven by exosomal lncRNA. Front. Genet..

[73] ElMonier A.A., El-Boghdady N.A., Fahim S.A., Sabry D., Elsetohy K.A., Shaheen A.A. (2023). LncRNA NEAT1 and MALAT1 are involved in polycystic ovary syndrome pathogenesis by functioning as competing endogenous RNAs to control the expression of PCOS-related target genes. Noncoding RNA Res..

[74] Ma Y., Ma L., Cao Y., Zhai J. (2021). Construction of a ceRNA-based lncRNA-mRNA network to identify functional lncRNAs in polycystic ovarian syndrome. Aging.

[75] Zhang R., Zhou Z., Wang P., He X., Liu Y., Chu M. (2024). The SLC19A1-AS/miR-1343/WNT11 axis is a novel positive regulatory ceRNA network governing goat granulosa cell proliferation. Int. J. Biol. Macromol..

[76] Wang M., Wang Y., Yang L., Du X., Li Q. (2023). Nuclear lncRNA NORSF reduces E2 release in granulosa cells by sponging the endogenous small activating RNA miR-339. BMC Biol..

[77] Liu B., Liu L., Sulaiman Z., Wang C., Wang L., Zhu J., Liu S., Cheng Z. (2024). Comprehensive analysis of lncRNA-miRNA-mRNA ceRNA network and key genes in granulosa cells of patients with biochemical primary ovarian insufficiency. J. Assist. Reprod. Genet..

[78] Jain N., Gupta P., Sahoo S., Mallick B. (2022). Non-coding RNAs and their cross-talks impacting reproductive health of women. Wiley Interdiscip. Rev. RNA.

[79] Lin N., Lin J.Z. (2022). Identification of long non-coding RNA biomarkers and signature scoring, with competing endogenous RNA networks- targeted drug candidates for recurrent implantation failure. Hum. Fertil..

[80] Hu H., Jia Q., Xi J., Zhou B., Li Z. (2020). Integrated analysis of lncRNA, miRNA and mRNA reveals novel insights into the fertility regulation of large white sows. BMC Genomics.

[81] Alfeghaly C., Castel G., Cazottes E., Moscatelli M., Moinard E., Casanova M., Boni J., Mahadik K., Lammers J., Freour T. (2024). XIST dampens X chromosome activity in a SPEN-dependent manner during early human development. Nat. Struct. Mol. Biol..

[82] Zhou M., Liu X., Qiukai E., Shang Y., Zhang X., Liu S., Zhang X. (2021). Long non-coding RNA Xist regulates oocyte loss via suppressing miR-23b-3p/miR-29a-3p maturation and upregulating STX17 in perinatal mouse ovaries. Cell Death Dis..

[83] Avner R., Wahrman J., Richler C., Ayoub N., Friedmann A., Laufer N., Mitrani-Rosenbaum S. (2000). X inactivation-specific transcript expression in mouse oocytes and zygotes. Mol. Hum. Reprod..

[84] Wu R., Li J., Li J., Zhang N., Zhou W., Ren L., Chen Q., Li Y. (2021). Construction of Competing Endogenous RNA Networks Incorporating Transcription Factors to Reveal Differences in Granulosa Cells from Patients with Endometriosis. Genet. Test. Mol. Biomarkers.

[85] Liu M., Zhu H., Li Y., Zhuang J., Cao T., Wang Y. (2020). Expression of serum lncRNA-Xist in patients with polycystic ovary syndrome and its relationship with pregnancy outcome. Taiwan J. Obstet. Gynecol..

[86] Radhakrishnan R., Kowluru R.A. (2021). Long Noncoding RNA MALAT1 and Regulation of the Antioxidant Defense System in Diabetic Retinopathy. Diabetes.

[87] Zeng R., Zhang R., Song X., Ni L., Lai Z., Liu C., Ye W. (2018). The long non-coding RNA MALAT1 activates Nrf2 signaling to protect human umbilical vein endothelial cells from hydrogen peroxide. Biochem. Biophys. Res. Commun..

[88] Li Y., Xiang Y., Song Y., Zhang D., Tan L. (2022). MALAT1 downregulation is associated with polycystic ovary syndrome via binding with MDM2 and repressing P53 degradation. Mol. Cell. Endocrinol..

[89] Sun L., Zhang P., Lu W. (2021). lncRNA MALAT1 Regulates Mouse Granulosa Cell Apoptosis and 17beta-Estradiol Synthesis via Regulating miR-205/CREB1 Axis. Biomed. Res. Int..

[90] Tu M., Wu Y., Wang F., Huang Y., Qian Y., Li J., Lv P., Ying Y., Liu J., Liu Y. (2022). Effect of lncRNA MALAT1 on the Granulosa Cell Proliferation and Pregnancy Outcome in Patients with PCOS. Front. Endocrinol..

[91] Wu L., Tu Z., Bao Y., Zhai Q., Jin L. (2022). Long noncoding RNA NEAT1 decreases polycystic ovary syndrome progression via the modulation of the microRNA-324-3p and BRD3 axis. Cell Biol. Int..

[92] Nakagawa S., Shimada M., Yanaka K., Mito M., Arai T., Takahashi E., Fujita Y., Fujimori T., Standaert L., Marine J.C. (2014). The lncRNA Neat1 is required for corpus luteum formation and the establishment of pregnancy in a subpopulation of mice. Development.

[93] Liu Y.X., Ke Y., Qiu P., Gao J., Deng G.P. (2023). LncRNA NEAT1 inhibits apoptosis and autophagy of ovarian granulosa cells through miR-654/STC2-mediated MAPK signaling pathway. Exp. Cell Res..

[94] Carbonell T., Gomes A.V. (2020). MicroRNAs in the regulation of cellular redox status and its implications in myocardial ischemia-reperfusion injury. Redox Biol..

[95] Bu H., Wedel S., Cavinato M., Jansen-Durr P. (2017). MicroRNA Regulation of Oxidative Stress-Induced Cellular Senescence. Oxid. Med. Cell. Longev..

[96] Ashrafizadeh M., Ahmadi Z., Samarghandian S., Mohammadinejad R., Yaribeygi H., Sathyapalan T., Sahebkar A. (2020). MicroRNA-mediated regulation of Nrf2 signaling pathway: Implications in disease therapy and protection against oxidative stress. Life Sci..

[97] Santonocito M., Vento M., Guglielmino M.R., Battaglia R., Wahlgren J., Ragusa M., Barbagallo D., Borzì P., Rizzari S., Maugeri M. (2014). Molecular characterization of exosomes and their microRNA cargo in human follicular fluid: Bioinformatic analysis reveals that exosomal microRNAs control pathways involved in follicular maturation. Fertil. Steril..

[98] Battaglia R., Vento M.E., Ragusa M., Barbagallo D., La Ferlita A., Di Emidio G., Borzí P., Artini P.G., Scollo P., Tatone C. (2016). MicroRNAs Are Stored in Human MII Oocyte and Their Expression Profile Changes in Reproductive Aging. Biol. Reprod..

[99] Battaglia R., Vento M.E., Borzì P., Ragusa M., Barbagallo D., Arena D., Purrello M., Di Pietro C. (2017). Non-coding RNAs in the Ovarian Follicle. Front. Genet..

[100] Battaglia R., Musumeci P., Ragusa M., Barbagallo D., Scalia M., Zimbone M., Lo Faro J.M., Borzì P., Scollo P., Purrello M. (2020). Ovarian aging increases small extracellular vesicle CD81+ release in human follicular fluid and influences miRNA profiles. Aging.

[101] Amin M.M.J., Trevelyan C.J., Turner N.A. (2021). MicroRNA-214 in Health and Disease. Cells.

[102] Battaglia R., Caponnetto A., Caringella A.M., Cortone A., Ferrara C., Smirni S., Iannitti R., Purrello M., D’Amato G., Fioretti B. (2022). Resveratrol Treatment Induces Mito-miRNome Modification in Follicular Fluid from Aged Women with a Poor Prognosis for In Vitro Fertilization Cycles. Antioxidants.

[103] Chang W., Wang J., Tao D., Zhang Y., He J., Shi C. (2016). Identification of a novel miRNA from the ovine ovary by a combinatorial approach of bioinformatics and experiments. J. Vet. Med. Sci..

[104] Zhang X., Dong C., Yang J., Li Y., Feng J., Wang B., Zhang J., Guo X. (2021). The Roles of the miRNAome and Transcriptome in the Ovine Ovary Reveal Poor Efficiency in Juvenile Superovulation. Animals.

[105] Zhou J., Jin X., Sheng Z., Zhang Z. (2021). miR-206 serves an important role in polycystic ovary syndrome through modulating ovarian granulosa cell proliferation and apoptosis. Exp. Ther. Med..

[106] Gad A., Sanchez J.M., Browne J.A., Nemcova L., Laurincik J., Prochazka R., Lonergan P. (2020). Plasma extracellular vesicle miRNAs as potential biomarkers of superstimulatory response in cattle. Sci. Rep..

[107] Zhang Z., Sang M., Liu S., Shao J., Cai Y. (2021). Differential expression of long non-coding RNA Regulator of reprogramming and its molecular mechanisms in polycystic ovary syndrome. J. Ovarian. Res..

[108] Diaz M., Bassols J., Lopez-Bermejo A., de Zegher F., Ibanez L. (2020). Low Circulating Levels of miR-451a in Girls with Polycystic Ovary Syndrome: Different Effects of Randomized Treatments. J. Clin. Endocrinol. Metab..

[109] Lu T., Zou X., Liu G., Deng M., Sun B., Guo Y., Liu D., Li Y. (2020). A Preliminary Study on the Characteristics of microRNAs in Ovarian Stroma and Follicles of Chuanzhong Black Goat during Estrus. Genes.

[110] De Nardo Maffazioli G., Baracat E.C., Soares J.M., Carvalho K.C., Maciel G.A.R. (2022). Evaluation of circulating microRNA profiles in Brazilian women with polycystic ovary syndrome: A preliminary study. PLoS ONE.

[111] Ibrahim S., Taqi M.O., Sosa A.S.A., El-Naby A.A.H., Mahmoud K.G.M., Darwish H.R.H., Abd El Hameed A.R., Nawito M.F. (2022). Spatiotemporal expression pattern of miR-205, miR-26a-5p, miR-17-5p, let-7b-5p, and their target genes during different stages of corpus luteum in Egyptian buffaloes. J. Genet. Eng. Biotechnol..

[112] Kim Y.Y., Kim K.S., Kim Y.J., Kim S.W., Kim H., Ku S.Y. (2021). Transcriptome Analyses Identify Potential Key microRNAs and Their Target Genes Contributing to Ovarian Reserve. Int. J. Mol. Sci..

[113] Wu H., Fan F., Liang C., Zhou Y., Qiao X., Sun Y., Jiang Y., Kang L. (2019). Variants of pri-miR-26a-5p polymorphisms are associated with values for chicken egg production variables and affects abundance of mature miRNA. Anim. Reprod. Sci..

[114] Kang L., Yang C., Wu H., Chen Q., Huang L., Li X., Tang H., Jiang Y. (2017). miR-26a-5p Regulates TNRC6A Expression and Facilitates Theca Cell Proliferation in Chicken Ovarian Follicles. DNA Cell Biol..

[115] Li Y., Wu X., Miao S., Cao Q. (2022). MiR-383-5p promotes apoptosis of ovarian granulosa cells by targeting CIRP through the PI3K/AKT signaling pathway. Arch. Gynecol. Obstet..

